# Nanoscale characterization of electrical transport at metal/3C-SiC interfaces

**DOI:** 10.1186/1556-276X-6-120

**Published:** 2011-02-07

**Authors:** Jens Eriksson, Fabrizio Roccaforte, Sergey Reshanov, Stefano Leone, Filippo Giannazzo, Raffaella LoNigro, Patrick Fiorenza, Vito Raineri

**Affiliations:** 1 CNR-IMM, Strada VIII n. 5, Zona Industriale, 95121, Catania, Italy; 2 Scuola Superiore-Università di Catania, Via San Nullo 5/i, Catania, 95123, Italy; 3 Acreo AB, Electrum 236, Kista, 16440, Sweden; 4 Department of Physics, Chemistry and Biology, Linköping University, Linköping, 58183, Sweden

## Abstract

In this work, the transport properties of metal/3C-SiC interfaces were monitored employing a nanoscale characterization approach in combination with conventional electrical measurements. In particular, using conductive atomic force microscopy allowed demonstrating that the stacking fault is the most pervasive, electrically active extended defect at 3C-SiC(111) surfaces, and it can be electrically passivated by an ultraviolet irradiation treatment. For the Au/3C-SiC Schottky interface, a contact area dependence of the Schottky barrier height (Φ_B_) was found even after this passivation, indicating that there are still some electrically active defects at the interface. Improved electrical properties were observed in the case of the Pt/3C-SiC system. In this case, annealing at 500°C resulted in a reduction of the leakage current and an increase of the Schottky barrier height (from 0.77 to 1.12 eV). A structural analysis of the reaction zone carried out by transmission electron microscopy [TEM] and X-ray diffraction showed that the improved electrical properties can be attributed to a consumption of the surface layer of SiC due to silicide (Pt_2_Si) formation. The degradation of Schottky characteristics at higher temperatures (up to 900°C) could be ascribed to the out-diffusion and aggregation of carbon into clusters, observed by TEM analysis.

## Introduction

With respect to the hexagonal silicon carbide polytype (4H-SiC), cubic silicon carbide (3C-SiC) has potential advantages for power device applications, specifically in terms of higher electron mobility in metal oxide semiconductor field-effect transistor [MOSFET] channels and due to the absence of bipolar degradation upon electrical stress [1,2]. However, the low stacking fault [SF] formation energy for this polytype [3] leads to high densities of these defects, which prevent the achievement of the predicted electrical properties. While 3C-SiC MOSFETs with excellent on-state characteristics have been demonstrated [4], a significant reduction of the SF density is required in order to achieve acceptable blocking voltage values and off-state leakage currents. Hence, it is crucial to better understand which role these defects play in the non-ideal contact properties and explore ways to mitigate their detrimental influence on devices. In this sense, imaging the evolution of the structural and electrical properties at SiC interfaces at a nanoscale level can be the way to better understand the physical transport phenomena and to finally improve the performance of potential devices.

In this work, the transport properties of metal/3C-SiC interfaces were characterized employing nanoscale techniques in combination with the conventional electrical measurements. In particular, the nanoscale electrical activity of SFs in the semiconductor at the contact interface was studied by conductive atomic force microscopy [C-AFM]. The effects of a surface passivation on the localized leakage currents through SFs and the characteristics of fabricated Au/-3C-SiC diodes were discussed. Also, the electrical and structural properties of the Pt/3C-SiC system were studied, where high-temperature annealing can induce metal/SiC interface reactions and strongly affect the barrier homogeneity and leakage currents caused by semiconductor surface states.

## Experimental

3C-SiC(111) heteroepitaxial 12-μm n-type ([N] = 2 × 10^17 ^cm^−3^) layers were grown onto (0001) 4H-SiC substrates using a chlorine-based chemical vapor deposition [CVD] process [5] (sample A). Homoepitaxial 4-μm n-type ([N] = 3 × 10^15 ^cm^−3^) 3C-SiC layers were grown on free-standing 3C-SiC(001) substrates [6] using CVD (sample B). Ohmic back contacts were formed by evaporation of a 100-nm-thick layer of Ni and a subsequent rapid annealing step at 950°C for 60 s to form nickel silicide [7,8]. The 3C-SiC surfaces were then subjected to a UV irradiation treatment at 200°C in order to electrically passivate carbon-related defect states at the surface [9,10]. Front contacts were formed by sputter deposition of a 100-nm-thick Au (samples A and B) or Pt film (sample B), and circular contacts with radii from 5 to 150 μm were defined by a lift-off process. The fabricated Pt diodes were annealed for 5 min at 500°C, 700°C, and 900°C in argon ambient. The structural evolution of the Pt/3C-SiC system upon thermal annealing was studied by X-ray diffraction [XRD] and transmission electron microscopy [TEM], which was also used to study structural defects in the 3C-SiC epilayers. XRD patterns were measured using a Bruker-AXS D5005 *θ*-*θ *diffractometer. TEM analyses were carried out after mechanical thinning and a final ion milling at low energy (5 keV Ar ions). Bright field images were recorded at 2-20 kV by a JEOL 2010F microscope equipped with the Gatan imaging filter. The effects of the structural evolution on the electrical behavior of fabricated Schottky diodes were investigated by current-voltage (*I*-*V*) characterization in a Karl Süss electrical probe station, as well as by C-AFM [11], performed using a Veeco DI dimension 3100, equipped with the nanoscope V electronics and the scanning spreading resistance [SSRM] module [12]. The parameters describing the metal/3C-SiC interfaces were extracted from *I*-*V *measurements using thermionic emission theory [13].

## Results and discussion

A key factor for the conduction properties through a contact is the quality of the metal/semiconductor interface, e.g., structural defects reaching the semiconductor surface can lead to a localized barrier lowering and increased leakage currents [14,15]. It has recently been shown by SSRM mapping, performed in cross-section, that extended defects in 3C-SiC cause a localized lowering of the resistance through the layer [16]. As a consequence, the crystalline quality of the 3C-SiC epilayer is pivotal for good device operation.

**Figure 1:**
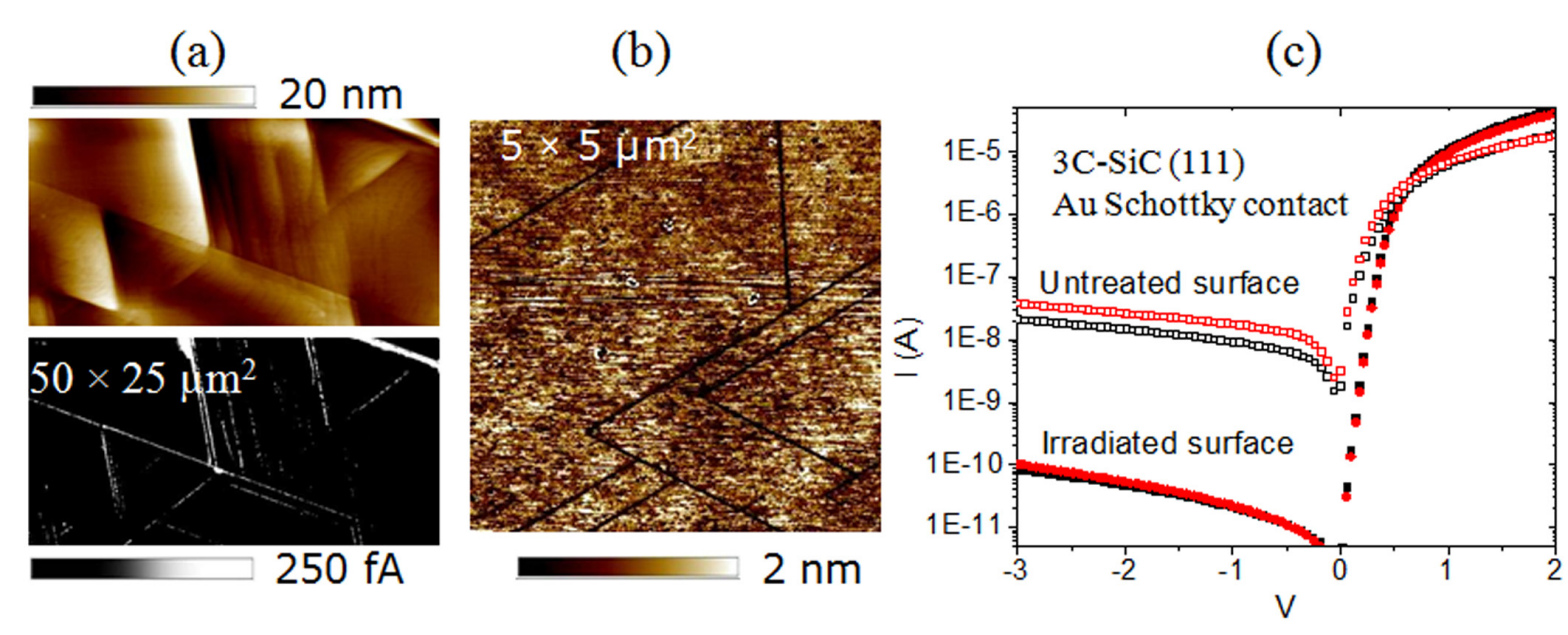
**Passivation of localized leakage currents, passing through SFs at the as-grown 3C-SiC surface, by UV irradiation**. C-AFM morphology (*top*) and current maps (*bottom*) of an as-grown 3C-SiC(111) surface at a tip bias of −2 V (**a**) showing localized leakage currents passing through stacking faults. The AFM morphology of the UV-irradiated 3C-SiC surface after a wet oxide etch revealed trenches in the SFs, suggesting that their passivation is due to a local oxidation at these defects. The *I*-*V *characteristics measured on Au/3C-SiC diodes with a contact radius of 20 μm exhibited greatly reduced leakage currents after passivation (**c**).

The micrographs in Figure 1a, obtained by applying a bias voltage of −2 V to the C-AFM tip that is scanned in contact mode on the semiconductor surface, show the morphology and the corresponding current map of the as-grown 3C-SiC(111) surface. As can be seen, leakage current preferentially flows through SFs, and several current maps determined on different areas on the sample alongside plan-view TEM analysis (not shown here, see, e.g., [9]) showed these to be the most pervasive extended defects affecting the electrical properties at the 3C-SiC surface and, hence, at the contact interface. In contrast, similar current maps measured after a UV surface treatment (as described in "Experimental") showed no localized leakage current through the SFs above the detection limit (*I *< 50 fA). Consequently, the electrical conduction through SFs at the 3C-SiC(111) surface can be suppressed by UV irradiation, during which ozone is generated. UV ozone treatment is known to remove surface defects in SiC related to carbon atoms due to oxidation [10], and SFs arriving at the 3C-SiC(111) have a C termination [17]. Indeed, the AFM morphology map in Figure 1b, obtained on the UV-irradiated 3C-SiC surface after selective wet oxide etching, reveals trenches of a few nanometers at the SF locations. Hence, the passivation of the SFs may result from a preferential oxidation occurring locally inside these defects where the polarity is shifted with respect to the Si-terminated (111) surface [17].

The effect of the passivation on the *I*-*V *behavior of fabricated Au/3C-SiC(111) diodes is shown in Figure 1c. A strong reduction of the leakage current is observed, while the forward current is largely unaffected. However, even after this passivation, a contact area dependence of the Schottky barrier height was observed for the Au/3C-SiC system where Φ_B _gradually increased from 0.7 to 1.4 eV as the contact radius was reduced from 150 to 5 μm [18].

The contact area dependence of Φ_B _observed for the Au/3C-SiC(111) system may result from an inhomogeneous interface between the metal and the semiconductor, or it could be caused by leakage currents related to surface states. Independent of its origin, this dependence should be mitigated if a 'fresh' metal/SiC interface is created instead of forming the contact interface at the original sample surface which was exposed to chemicals, sputter damage, and to air.

Pt is known to react with SiC at high temperatures, thus consuming a thin surface layer of SiC to form platinum silicide, in turn forming a new interface with the SiC. Both Pt and its silicides have high work functions that should result in good barrier heights on 3C-SiC [19]. Hence, we investigated the properties of the Pt/3C-SiC system upon high-temperature annealing. Previous studies on Pt contacts to 3C-SiC have reported the onset of platinum silicide phase formation to occur at annealing temperatures ranging from 650°C to above 750°C [20,21]. Moreover, both increased [22] and reduced [21,23] leakage currents have been reported upon silicide phase formation. Clearly, the effects of high-temperature annealing on the structural and electrical properties of Pt/3C-SiC is ambiguous. In this study, XRD analysis (not reported here) showed that the Pt_2_Si phase was formed already after annealing the Pt/3C-SiC(001) system at 500°C. The Pt_2_Si phase is thermodynamically stable in the studied temperature range [22], and the XRD patterns remain essentially the same also after annealing at 700°C and 900°C. Ternary compounds are not stable, meaning that carbon must be freed during the reaction. Indeed, the XRD spectra showed an increased presence of crystalline carbon with increasing annealing temperature.

**Figure 2:**
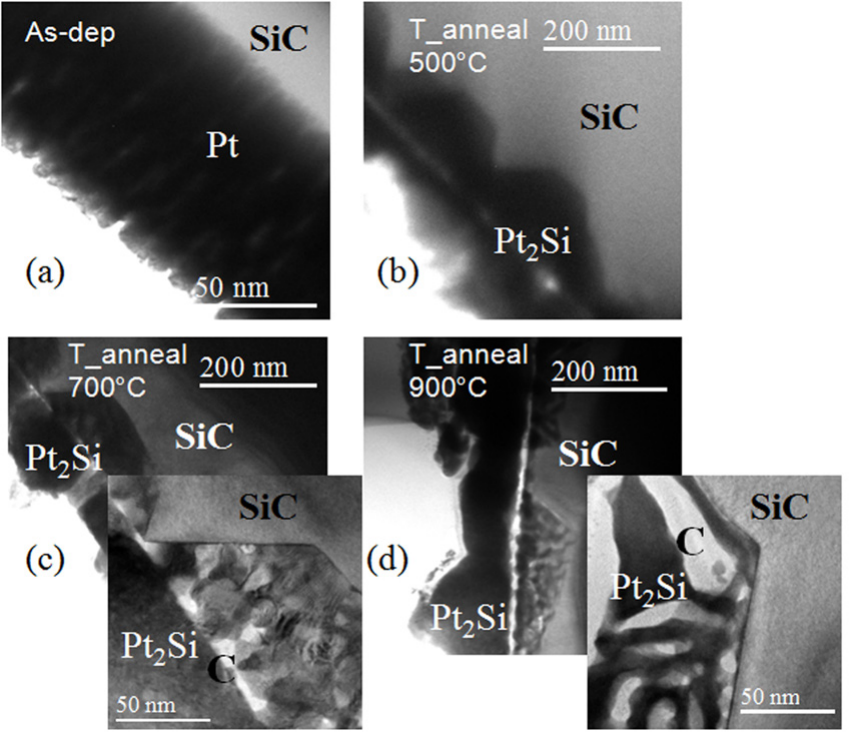
**TEM images of the Pt(Pt_2_Si)/SiC interface**. Bright-field, cross-section TEM images of the Pt(Pt_2_Si)/3C-SiC interface for the as-deposited Pt (**a**) and after annealing at 500°C (**b**), 700°C (**c**), and 900°C (**d**).

**Figure 3:**
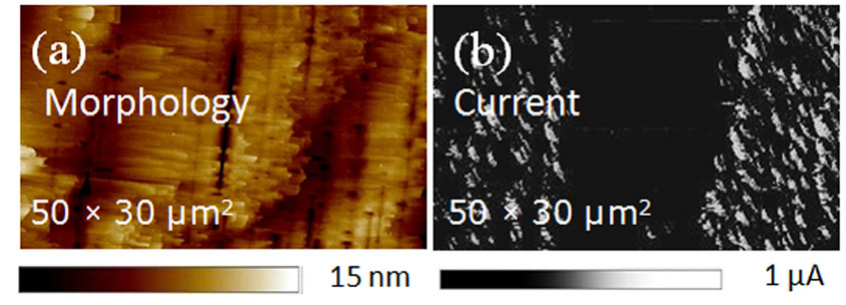
**Morphology of the SiC surface and current map of an adjacent Pt contact**. AFM morphology of the 3C-SiC(001) surface (**a**) and C-AFM current map determined at a tip bias of −5 V on an adjacent Pt contact after annealing at 500°C (**b**).

While XRD coupled with TEM analysis (see Figure 2) showed that all the Pt have been converted into the stable Pt_2_Si phase already at 500°C, higher temperature annealing gives rise to increased localized high leakage current areas at the contact interface. Figure 3a shows the morphology of the SiC surface where the large vertical lines are due to several stacking faults bunching together during the growth of the 3C-SiC substrate. Comparing Figure 3a with the current map of an adjacent Pt contact (Figure 3b) determined in the same sample orientation, it is clear that the localized leakage spots occur preferentially along the direction of stacking faults. The total area that is covered by these leaky spots was determined from current maps measured after each annealing temperature and increases from 12% at 500°C to 28% and 55% after annealing at 700°C and 900°C, respectively. These leakage spots suggest a Schottky barrier inhomogeneity, characterized by local low-barrier patches of about 0.5-1.5 μm in diameter. Clearly, the evolution of these low-barrier patches will affect the properties of the fabricated diodes. Indeed, the existence of low-barrier patches contributing to an overall lowering of the average barrier is a common way of modeling non-ideal macroscale diode behavior [24].

**Figure 4:**
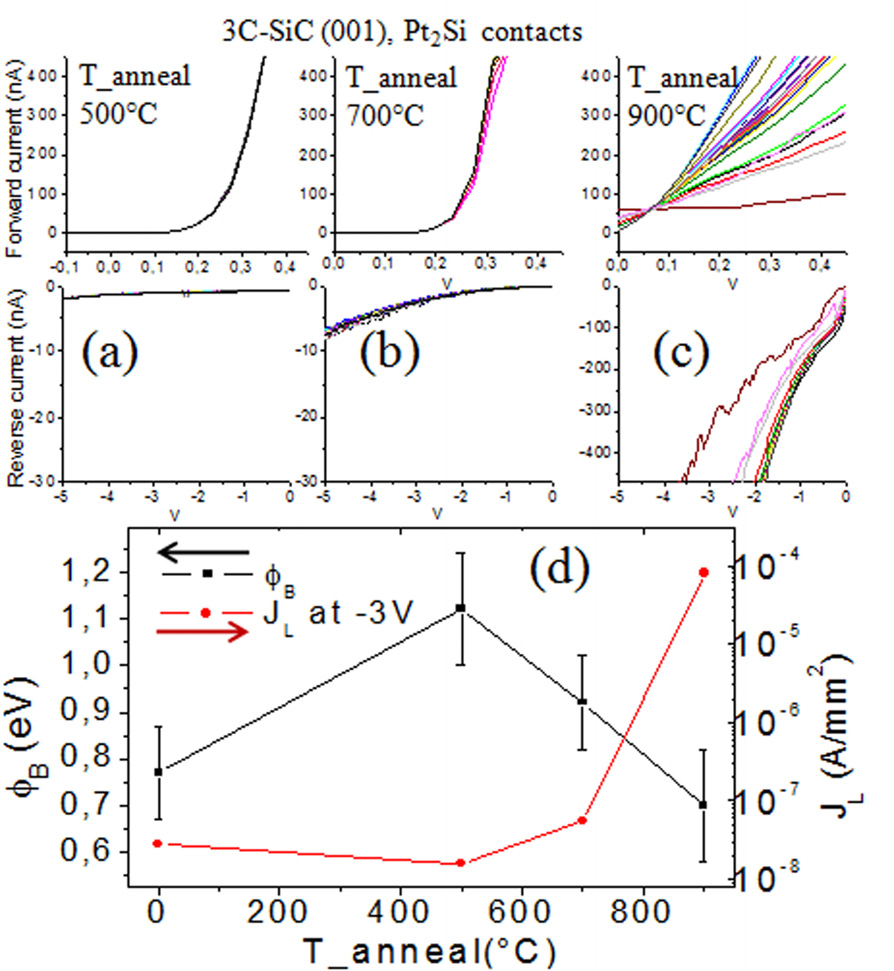
***I*-*V *spectroscopy, measured by C-AFM, and corresponding diode parameters**. Localized forward (*top*) and reverse (*bottom*) *I*-*V *spectroscopy measured by C-AFM at 25 different tip locations on the Pt_2_Si contacts after annealing at 500°C (**a**), 700°C (**b**), and 900°C (**c**). The Schottky barrier heights (*black*, *left axis*) and leakage current densities taken at −3 V (*red*, *right axis*) extracted from *I*-*V *probe measurements for the different annealing temperatures are shown in (**d**).

Figure 4 shows localized *I*-*V *spectroscopy measured by C-AFM at 25 different tip locations (separated by 1 μm) on the Pt_2_Si contacts after annealing at 500°C (a), 700°C (b), and 900°C (c). Increased local variations are observed under both forward (barrier height) and reverse (leakage currents) bias as the annealing temperature increases, consistent with the increasing presence of localized low-barrier patches at the contact interface. *I*-*V *characteristics were also measured in an electrical probe after each annealing step on circular diodes with radii of 20 and 100 μm, and the extracted diode parameters are summarized in Figure 4d. Already, the as-deposited Pt/3C-SiC(001) contacts exhibited improved electrical properties with respect to the Au/3C-SiC(001) system; the leakage current density (at −3 V) measured for Au and Pt diodes fabricated on the same wafer (sample B) reduced from 1 × 10^−6 ^to 3 × 10^−8 ^A/mm^2^. Moreover, the contact area dependence observed for the Au/3C-SiC system is absent for the Pt/SiC interface, suggesting better interface homogeneity. As can be seen in Figure 4d, the leakage current is slightly improved after annealing at 500°C compared to the as-deposited Pt, whereas a strong improvement of the Schottky barrier height (from 0.77 to 1.12 eV) is observed for this annealing temperature. At higher temperatures (700°C and 900°C), both the reverse and forward *I*-*V *characteristics begin to degrade.

To understand the electrical evolution upon annealing, the cross-section of the contact interface was studied by TEM. As seen in the TEM images in Figure 2 the Pt layer (Figure 2a) has reacted completely after the 500°C anneal (Figure 2b), and the consumption of Pt results in the formation of a polycrystalline Pt_2_Si layer, characterized by the presence of interfacial protrusions penetrating into the underlying SiC [22]. However, additional changes are observed at the higher temperatures. At 700°C (Figure 2c), larger size variations are visible in the Pt_2_Si protrusions and there is a formation of a carbon layer at the interface and carbon clusters inside the protrusions (also confirmed by energy-filtered TEM). After annealing at 900°C, this layer is thicker and more pronounced carbon clusters are observable inside the protrusions.

The TEM observations can be consistently correlated to the previously shown electrical results. Since Pt_2_Si is the only phase observed by XRD for the different annealing temperatures, the evolution of the electrical properties after annealing above 500°C is likely unrelated to the silicide phase formation. More likely, the changes in the electrical properties can be related to changes in the density of states at the contact interface. As reported by Mullins and Brunnschweiler [25], for the as-deposited contacts, the surface states are presumed to behave as donor-like traps, generated during the sputtering of the metal. Hence, after annealing at 500°C, the interface moves away from the original 3C-SiC surface and these states no longer affect the properties of the contact, causing a widening of the energy barrier that in turn causes the tunneling leakage current to reduce. The gradual degradation observed at higher annealing temperatures can be explained by the increased amount of carbon clusters at the contact interface and in the Pt_2_Si protrusions due to incomplete diffusion of carbon upon further consumption of SiC, which has been shown to become an issue at annealing above 600°C [23]. The agglomeration of electrically active carbon clusters at the interface gives rise to barrier inhomogeneities, characterized by local low-barrier patches, ultimately leading to an increase of the leakage current [26].

## Conclusion

Nanoscale electrical and structural characterization of metal/3C-SiC interfaces were performed using C-AFM, XRD, and TEM. It was shown that the most pervasive extended defect at 3C-SiC(111) surfaces, the stacking fault, can be passivated by UV irradiation treatment. This allowed an almost ideal Schottky behavior to be measured for the Au/3C-SiC system when characterizing very small diodes. However, a contact area dependence of the Schottky barrier height was found after this passivation, indicating that other electrically active defects and/or inhomogeneities at the contact interface still remain even after UV passivation. The contact area dependence of Φ_B _was absent for the Pt/3C-SiC system, which also showed improved electrical properties with respect to the Au/3C-SiC system. Annealing of Pt/3C-SiC at 500°C resulted in further reduction of the leakage current and an increase of the Schottky barrier height. These changes are attributed to a consumption of the surface layer of SiC due to Pt_2_Si formation. However, upon annealing at higher temperatures (700°C and 900°C), a degradation of the Schottky characteristics occurred. TEM analysis showed that this degradation can be ascribed to the aggregation of carbon clusters at the interface.

## Competing interests

The authors declare that they have no competing interests.

## Authors' contributions

JE designed the experiments, carried out the sample preparation, performed the electrical measurements and drafted the manuscript. FG and PF supported JE during scanning probe microscopy measurements; RLN performed the XRD measurements and analysis; SR and SL provided the 3C-SiC samples. FR and VR coordinated the research activity and helped design the experiments. All authors took part in the discussion of the results and helped shape the final manuscript. All authors read and approved the final manuscript
